# Beringian Standstill and Spread of Native American Founders

**DOI:** 10.1371/journal.pone.0000829

**Published:** 2007-09-05

**Authors:** Erika Tamm, Toomas Kivisild, Maere Reidla, Mait Metspalu, David Glenn Smith, Connie J. Mulligan, Claudio M. Bravi, Olga Rickards, Cristina Martinez-Labarga, Elsa K. Khusnutdinova, Sardana A. Fedorova, Maria V. Golubenko, Vadim A. Stepanov, Marina A. Gubina, Sergey I. Zhadanov, Ludmila P. Ossipova, Larisa Damba, Mikhail I. Voevoda, Jose E. Dipierri, Richard Villems, Ripan S. Malhi

**Affiliations:** 1 Department of Evolutionary Biology, University of Tartu, Estonian Biocentre, Tartu, Estonia; 2 Leverhulme Centre for Human Evolutionary Studies, University of Cambridge, Cambridge, United Kingdom; 3 Department of Anthropology, University of California at Davis, Davis, California, United States of America; 4 Department of Anthropology, University of Florida, Gainesville, Florida, United States of America; 5 Instituto Multidisciplinario de Biología Celular, La Plata, Argentina; 6 Department of Biology, University of Rome Tor Vergata, Rome, Italy; 7 Institute of Biochemistry and Genetics, Ufa Research Center, Russian Academy of Sciences, Ufa, Russia; 8 Department of Molecular Genetics, Yakut Research Center, Russian Academy of Medical Sciences, Yakutia, Russia; 9 Institute of Medical Genetics, Tomsk Research Center, Russian Academy of Medical Sciences, Tomsk, Russia; 10 Institute of Genetics and Cytology, Siberian Branch of Russian Academy of Sciences, Novosibirsk, Russia; 11 Department of Anthropology, University of Pennsylvania, Philadelphia, Pennsylvania, United States of America; 12 Instituto de Biologia de la Altura–Universidad Nacional de Jujuy, Jujuy, Argentina; 13 Department of Anthropology, Institute for Genomic Biology, University of Illinois at Urbana-Champaign, Champaign, Illinois, United States of America; University of Sydney, Australia

## Abstract

Native Americans derive from a small number of Asian founders who likely arrived to the Americas via Beringia. However, additional details about the intial colonization of the Americas remain unclear. To investigate the pioneering phase in the Americas we analyzed a total of 623 complete mtDNAs from the Americas and Asia, including 20 new complete mtDNAs from the Americas and seven from Asia. This sequence data was used to direct high-resolution genotyping from 20 American and 26 Asian populations. Here we describe more genetic diversity within the founder population than was previously reported. The newly resolved phylogenetic structure suggests that ancestors of Native Americans paused when they reached Beringia, during which time New World founder lineages differentiated from their Asian sister-clades. This pause in movement was followed by a swift migration southward that distributed the founder types all the way to South America. The data also suggest more recent bi-directional gene flow between Siberia and the North American Arctic.

## Introduction

The mitochondrial DNA haplogroup nomenclature that is widely used today in population and medical genetics, forensic science, and in other interdisciplinary studies, traces back to the analysis of Native American populations by Torroni et al. [1],[2]. The first four letters of the phylogenetic alphabet for mtDNA haplogroups - A-D - were coined to refer to just four founding haplogroups that exhibit virtually all North and South American mtDNA diversity.

Genetic studies demonstrate that Native Americans inherited their mitochondrial DNA (mtDNA) from a handful of founders who arrived from Asia *via* Beringia [1],[2]. No more than four major pan American and three minor North American founding mtDNA haplotypes (A2, B2, C1, D1 and X2a, D2, D3, respectively) have been convincingly established in previous studies of control region sequence, RFLP markers and 30 complete mtDNA genomes (Table 1) [1]–[14]. The paucity of established founding mtDNAs suggests that the number of migrants that initially peopled the Americas was relatively low. However, determining the full range of diversity surviving to the present day in the founding population requires high-resolution mtDNA sequence data. Previous estimates of mtDNA diversity are predominantly based on control region sequences representing only a minor fraction of the mtDNA genome. In addition, control region sequences experience a high frequency of recurrent mutations, potentially obscuring the identification of additional founding mtDNAs [14]–[19].

**Table 1:** Defining mutations for Native American mtDNA haplogroups

Hg	HVS I	HVS II	Coding region
A	16223-16290-16319	73-235-263	663, 1736, 4248, 4824, 8794
**A2**	**16111**-16223-16290-16319-16362	**64**-73-**146**-**153**-235-263	**8027**, **12007**
**A2a**	**16111**-16223-16290-16319-16362	**64**-73-**146**-**153**-235-263	**3330**
B	16189	73-263	8281-8289del
B4bd	16189-16217	73-263	827, 15535
B4b	16189-16217	73-263	499, 4820, 13590
** B2**	16189-16217	73-263	**3547**, **4977**, **6473**, **9950**, **11177**
C	16223-16298-16327	73-249d-263	3552A, 9545, 11914, 13263, 14318
** C1**	16223-16298-**16325**-16327	73-249d-263-**290-291d**	**-**
** C1b**	16223-16298-16325-16327	73-249d-263-290-291d	**493**
** C1c**	16223-16298-16325-16327	73-249d-263-290-291d	**1888**, **15930**
** C1d**	16223-16298-16325-16327	73-249d-263-290-291d	**7697**
C4	16223-16298-16327	73-249d-263	2232iA, 6026, 11969, 15204
** C4c**	16223-**16245**-16298-16327	73-263	**11440, 13368, 14433, 15148**
D	16223-16362	73-263	4883, 5178A
D4	16223-16362	73-263	3010, 8414, 14668
** D1**	16223-**16325**-16362	73-263	**2092**
** D2**	**16129**-16223-**16271**-16362	73-263	3316, **7493**, **8703**, 9536, 11215
** D2a**	**16129**-16223-**16271**-16362	73-263	**11959**
** D2b**	**16129**-16223-**16271**-16362	73-263	**9181**
** D4h3**	16223-**16241**-**16301**-**16342**-16362	73-263	3336, **3396**, 3644, 5048, **6285**, **8949**, **9458**, **13135**
** D3**	16223-**16319**-16362	73-263	**951**, 8020, 10181, 15440, 15951
X	16189-16223-16278	73-153-263	6221, 6371, 13966, 14470
** X2a**	16189-**16213-**16223-16278	73-153-195-**200**-263	1719, **8913**, **12397**, **14502**

The full substitutional motif is shown in control region, the sub-clades defining mutations are indicated in bold.

Even though some additional minor founder types have been later identified in North America, such as X, the hypothesis of just four major founder types in the initial colonization of the New World remains uncontested. However, the timing of their entry remains debated. Previous studies of mtDNA data place estimates for the peopling of the New World in a broad range from 11,000 to over 40,000 years before present (ybp) [reviewed by 20], although more recent estimates range from 20,000–15,000 ybp [21]. Recent archaeological evidence places *Homo sapiens* in northeastern Siberia at the Yana Rhinoceros Horn Site as early as 30,000 ybp [22] about twice the 15,000 ybp [23] date for humans at the southern end of South America. These archaeological dates suggest two likely scenarios. First, the ancestors of Native Americans peopled Beringia before the Last Glacial Maximum, but remained locally isolated (likely due to ecological barriers) until entering the Americas at 15,000 ybp (Beringian incubation model, BIM) [24]. Second, the ancestors of Native Americans did not reach Beringia until just before 15,000 ybp, and then moved continuously on into the Americas, being recently derived from a larger parent Asian population (direct colonization model, DCM).

The DCM model hypothesizes the presence of founding mtDNA haplotypes that include members from both Northeast Asia and the Americas. It presumes a continuous movement of recently derived migrants across Beringia. In contrast, the BIM model predicts widespread, derived founding haplotypes specific to the Americas that are not found in Asia. This implies that migrants were isolated for an extended period of time before entering the Americas and that the founder haplotypes arose *in situ* in Beringia. Once in the Americas, these immigrants spread southward. Therefore, the phylogeographic distribution of this diversity can provide insights into the mode of the initial phase of the peopling of the Americas. A nested hierarchy of diversity from north to south in Native American founding haplogroups would reflect a gradual peopling, whereas a uniform distribution of Native American founding haplotypes both in North and South America implies a rapid occupation.

## Results

**Figure 1:**
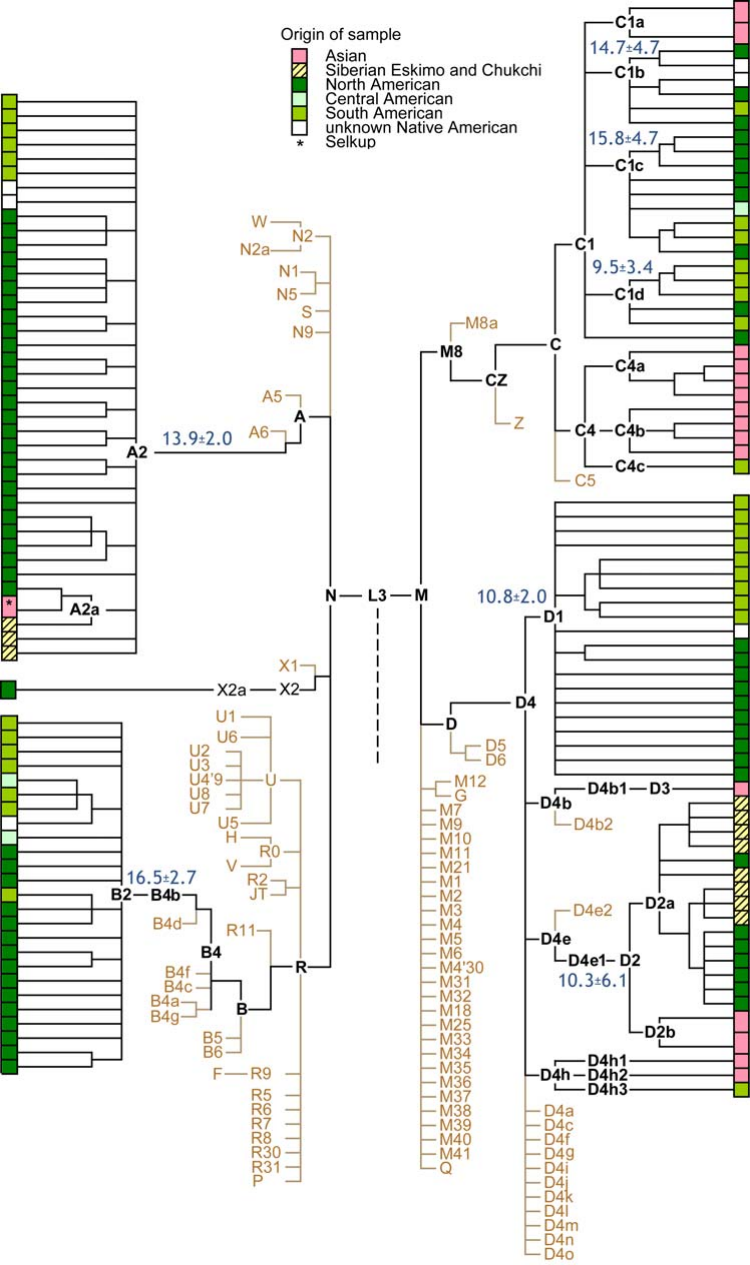
Schematic representation of phylogeny of human mtDNA outside of Africa. Branches encompassing Native Americans and their immediate Asian ancestral and sister lineages, represented by complete sequences, are shown in black with coalescence ages indicated and geographic location identified by colours. Lineages in brown correspond to the main haplogroups, found in Eurasia and Oceania, but absent in Native Americans. For complete phylogenetic tree see Supplementary figure 1.

Previous studies of mtDNA variation recognized no major subclade structure within each New World haplogroup [1],[3]–[4],[24]. A few studies of mtDNA variation suggested subclade structure [12],[14],[25], but lacked the power to convincingly demonstrate it. In this study we identified three sub-clades - C1b, C1c and C1d - that incorporate nearly all of Native American haplogroup C mtDNAs. All three are widely distributed in the New World. They are absent in Asia, and show similar coalescence times of approximately 13,900±2,700 years ago (Figure 1). Similar coalescence times were estimated for the other major founder haplogroups - A2, B2 and D1 - suggesting the simultaneous divergence of all founder clades across North and South America. A different C1 sub-clade in Asia - C1a [26]- likely derives from the same ancestral population as the three Native American sub-clades. Thus C1b, C1c and C1d are likely independent New World founders. In addition to C1 sub-clades, we defined two additional founders–D4h3 and C4c. These differ by several mutations from the Asian-derived ancestral clades, D4h and C4, respectively (Figure S1d,c). Haplogroup D4h3 ranges from Alaska to Tierra del Fuego and has recently been identified in Alaskan skeletal remains (10,300 ybp) [27]. We identified haplogroup C4c in two Ijka-speakers from Colombia, but its distribution in the Americas remains poorly characterized.

## Discussion

**Figure 2:**
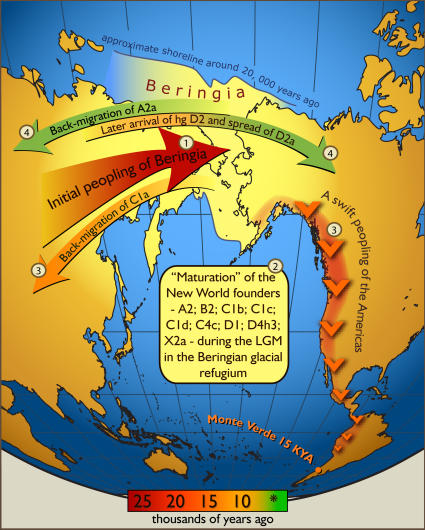
Schematic illustration of maternal geneflow in and out of Beringia. Colours of the arrows correspond to approximate timing of the events and are decoded in the coloured time-bar. The initial peopling of Berinigia (depicted in light yellow) was followed by a standstill after which the ancestors of the Native Americans spread swiftly all over the New World while some of the Beringian maternal lineages–C1a-spred westwards. More recent (shown in green) genetic exchange is manifested by back-migration of A2a into Siberia and the spread of D2a into north-eastern America that post-dated the initial peopling of the New World.

Our phylogeographic analysis of a new mitochondrial genome dataset allows us to draw several conclusions. First, before spreading across the Americas, the ancestral population paused in Beringia long enough for specific mutations to accumulate that separate the New World founder lineages from their Asian sister-clades (Figure 2) [4],[24], [28]–[29]. Second, founding haplotypes are uniformly distributed across North and South America instead of exhibiting a nested structure from north to south (Figure 1). Thus, after the Beringian standstill, the initial North to South migration was likely a swift pioneering process, not a gradual diffusion. This scenario matches the pattern of distribution of the first archaeological sites in Northeast Asia and the Americas [22],[23]. Third, the largely autochthonous pattern of variation seen in Native American mtDNAs suggests that the swift migration was followed by long-term isolation of local populations accompanied with the development of regional haplotypes within continental founder haplogroups [1].

In addition to illuminating the peopling process during the pioneering phase, the new dataset allows identification of more-recent genetic exchanges around and across Beringia (Figure 2). Specifically, haplogroup D2 consists of two sister clades, one found only in Siberia (D2b) and the other found in northernmost Eskimos, Chukchi, Aleut, and Athapaskans (D2a). While sub-haplogroup D2a is shared between ethno-historically close related Beringian Aleuts and Eskimos, (Figure S1) its sister clade D2b is spread among populations from distantly related linguistic groups (Tungusic, Turkic, Mongolic) (Table S2). A close relationship of matrilineal ancestry between individuals from different linguistic groups may be due to an overlap of geographic range of their ancestors approximately at the time of the Pleistocene-Holocene boundary. Alternatively, some populations may have received the D2b variant through more recent gene flow. It is also worthwhile to note the absence of D2 in all other Native American populations, suggesting that D2 diversified in Beringia after the initial migration into the Americas had occurred. Haplogroup D3 may have also reached America through more recent genetic exchange. It is spread in Nganasans, Mansi, Evenks, Ulchi, Tuvas, Chukchi and Siberian Eskimos [26],[30] and recently reported in Greenland and Canadian Inuit populations [31], but absent in other Native Americans. Additional investigatios of these populations may provide insight into the cause of the phylogenetic connections.

Surprisingly, we also found a Native American sub-type of haplogroup A2 among Evenks and Selkups in southern and western Siberia (Table S2). Previously, this HVS I motif is reported in one Yakut-speaking Evenk in northwestern Siberia [32]. A novel demographic scenario of relatively recent gene flow from Beringia to deep into western Siberia (Samoyedic-speaking Selkups) is the most likely explanation for the phylogeography of haplogroup A2a, which is nested within an otherwise exclusively Native American A2 phylogeny (Figure S1).

The high-resolution sequence data analyzed in this study reveals previously hidden diversity within the Native American mtDNA gene pool. The new data suggest that the initial founders of the Americas emerged from a single source ancestral population that evolved in isolation, likely in Beringia. This scenario is consistent with the unique pattern of diversity from autosomal locus D9S1120 [33] of a private allele in high frequency and ubiquitous in the Americas. The finding that humans were present at the Yana Rhinoceros Horn Site dated to 30,000 ybp [22] suggests that the isolation in Beringia might have lasted up to 15,000 years. Following this isolation, the initial founders of the Americas began rapidly populating the New World from North to South America.

## Materials and Methods

The sample-set comprises 601 Native American individuals from 20 populations distributed throughout the Americas (23 Dogribs from Subarctic Canada; 20 Apaches, 20 Northern Paiutes, 11 Zunis from Southwest US; 77 Ngöbes, 34 Kunas from Panama; 39 Emberas, 57 Waunanas from Panama and Colombia; 47 Arsarios, 48 Koguis, 29 Ijkas, 42 Wayuus, 27 Coreguajes, 22 Vaupes from Colombia; 12 Secoyas-Sionas, 32 Cayapas from Ecuador; 9 Tucuman, 18 Salta, 25 Catamarca, 5 Mocovi from Argentina) and 3764 samples from 26 Asian populations (51 Eskimos; 155 Chukchi; 120 Selkups; 66 Kets; 70 Tundra Nenets; 275 Tuvas; 185 Khakas; 339 Altaians; 170 Shors; 71 Koryaks; 85 Nanais; 122 Uyghurs; 406 Kazakhs; 58 Gilyaks; 61 Oroks; 105 Kirghiz; 48 Uzbeks; 38 Tajiks; 201 Buriats; 324 Evenks; 105 Evens; 22 Yukaghirs; 423 Yakuts; 157 Dolgans; 107 Nganasans). A subset of these sequences were reported elsewhere [34]–[42].

First, haplogroup affiliations of the individual samples were determined through RFLP analysis and DNA sequencing of the HVS I region, if not known earlier [34]–[42]. Samples that could not be assigned to haplogroups A–D or X were investigated for evidence of recent admixture, particularly among populations with well established historical accounts of co-existence of Native American and either European or African populations. Samples of European or African origin were excluded from the current study. Further, 20 Native American and 7 Asian samples were selected for complete sequencing of mtDNA genomes. Using these 27 novel and 113 published Native American and relevant Asian complete or coding region sequences [4],[26],[30],[43]–[50], phylogenetic trees were reconstructed based on a maximum parsimony approach (Figure S1, Text S1). From these whole mtDNA genomes coding region markers were selected for screening in the sample set through RFLP analyses or direct sequencing (Table S1). Hierarchical method was used, so, that each Native American sample was first cheked for nucleotide positions, where a polymorphism could be assumed based on the HVS I information and close ethnic, geographic or linquistic affiliation to complete sequenced sample. From Asian populations, samples, which could be relevant to Native American haplogroups, were selected based on HVS I sequence and analyzed for coding region markers (A2–12007, 8027; A2a–3330; C1a–3826, 7598; C4–11969; C4a–12672; C4b–3816; C4c–11440; D2–8703; D2a–4991, 11959; D2b–9181; D4–8414T; D4a–3206; D4e1–3316; D4h–3336, 3644; D4m–9667; D5–5301; D5a–11944, 12026).

DNA was extracted using conventional methods [34]–[42]. Preparation of sequencing templates was carried out following standard protocols, employing FIREPol polymerase (Solis BioDyne). Purified products were sequenced with the DYEnamic™ ET terminator cycle sequencing kit (Amersham Pharmacia Biotech) and analyzed on MegaBace1000 or ABI 3730xl sequencers. Sequences were aligned and analyzed with the Wisconsin Package (GCG) or ChromasPro 1.34.

Coalescence-age calculations and SDs were estimated based on the phylogenies of complete sequences [3],[6]. Given the global propensity of young mtDNA clades showing a significant excess of non-synonymous mutations, application of the raw molecular clock [49] in intra-species data sets is problematic [46]. Therefore, for dating the coalescent times of founder haplogroups we employed only synonymous transitions between the np 577-16023, assuming the rate of 3.5×10-8 (SD 0.1×10-8)/year/position [46]. The complete mtDNA genome data can be found in Genbank.

## Supporting Information

Figure S1 Complete Phylogenetic Trees
(0.10 MB XLS)

Text S1 Legend for Figure S1.
(0.03 MB DOC)

Table S1 Genotype data
(0.71 MB XLS)

Table S2 Haplogroup frequencies.
(0.02 MB XLS)
