# Supplementary material for: Beringian Standstill and Spread of Native American Founders
Source: PLoS One. 2007 Sep 5;2(9):e829. doi: 10.1371/journal.pone.0000829 (PMC1952074; doi:10.1371/journal.pone.0000829)
Supplement: Text S1 — Legend for Figure S1. (0.03 MB DOC) [file pone.0000829.s002.doc]

**Supplementary**

**Figure 1(a-d,x).**

Maximum parsimony tree of 140 complete mitochondrial DNA (mtDNA) sequences, given in five panels (a-e) presenting those belonging to haplogroups A (n=39), B (n=25), C (n=32), D (n=41) and X (n=3), respectively. 27 samples indicated in red are sequenced in the present study, 58 are from S1 and S2, 8 from S3, 2 from S4, 8 from S5, 13 from S6, 16 from S7, 1 from S8, 3 from S9, 2 from S10 and 1 from S11. The nucleotide substitutions are listed relative to the revised Cambridge reference sequence (S12) and are transitions unless a transversion is specified by a base change in capital letter. „del” denotes deletion, and „i” denotes insertion, „s” indicates synonymous substitution. The amino acid replacements are shown in parentheses. -t stands for change in a tRNA gene; -r, indicates a change in a rRNA gene. Recurrent mutations are underlined. Control region sequence is not reported in some published datasets (incl. S7 and samples belonging to hgs B (except 419), C and D in S1, S2) and the respective variation for the known samples is shown in italics. Point mutations at 16182 and 16183 were excluded because of their dependence on the presence of C-T transition at 16189 and mutations at 16519, 309 and 315 were omitted because of their hypervariability. The continental origin (when known) of the samples is indicated with colour.

In hg C, the samples 145, 417 and Am06 lack information for position 493, however, they share a mutation with sequences having a transition at 493 and were assigned to the C1b sub-clade as the most parsimonious solution. The sample 174 (S1) does not share any mutations with other sequences but could belong to sub-hg C1b.

For naming the new clades we followed the convincingly established Native American haplotypes and the existing haplogroup nomenclature (S11, S13-S17) and tried to resolve arising inconsistencies in the light of new data. The Asian sub-clade structure is mainly based on Kong et al. (S15).

Coalescence time estimates are shown next to clade labels and were calculated based on ρ (the average number of synonymous transitions to the root of the clade) (S13). A mutation rate of one synonymous transition per 6764 years between nps 577-16023 was used (S7).

**References**

S1. **Herrnstadt C, Elson JL, Fahy E, Preston G, Turnbull DM, et al.** (2002) Reduced-median-network analysis of complete mitochondrial DNA coding-region sequences for the major African, Asian, and European haplogroups. Am J Hum Genet 70:1152-1171.

S2. **Herrnstadt C, Preston G, Howell N.** (2003) Errors, phantoms and otherwise, in human mtDNA sequences. Am J Hum Genet 72:1585-6.

S3. **Mishmar D, Ruiz-Pesini E, Golik P, Macaulay V, Clark AG, et al.** (2003) Natural selection shaped regional mtDNA variation in humans. Proc Natl Acad Sci USA 100:171-176.

S4. **Maca-Meyer N, Gonzįlez AM, Larruga JM, Flores C, Cabrera VM.** (2001) Major genomic mitochondrial lineages delineate early human expansions. BMC Genet 2:13.

S5. **Ingman M, Kaessmann H, Pääbo S, Gyllensten U.** (2000) Mitochondrial genome variation and the origin of modern humans. Nature 408:708-713.

S6. **Derbeneva OA, Sukernik RI, Volodko NV, Hosseini SH, Lott MT, Wallace DC.** (2002) Analysis of mitochondrial DNA diversity in the Aleuts of the Commander Islands and its implications for the genetic history of Beringia. Am J Hum Genet 71:415-21.

S7. **Kivisild T, Shen P, Wall DP, Do B, Sung R, et al.** (2006) The role of selection in the evolution of human mitochondrial genomes. Genetics 172:373-87.

S8. **Kong Q-P, Yao Y-G, Sun C, Bandelt H-J, Zhu C-L, Zhang Y-P.** (2003) Phylogeny of East Asian mitochondrial DNA lineages inferred from complete sequences. Am J Hum Genet 73:671-676.

S9. **Starikovskaya EB, Sukernik RI, Derbeneva OA, Volodko NV, Ruiz-Pesini E.** (2005) Mitochondrial DNA diversity in indigenous populations of the southern extent of Siberia, and the origins of Native American haplogroups. Ann Hum Genet 69: 67-89.

S10.**Tanaka M, Cabrera VM, Gonzalez AM, Larruga JM, Takeyasu T, et al.** (2004) Mitochondrial genome variation in eastern Asia and the peopling of Japan. Genome Res 14:1832–1850.

S11.**Bandelt HJ, Hernnstadt C, Yao YG, Kong QP, Kivisild T, et al.** (2003) Identification of Native American founder mtDNas through the analysis of complete mtDNA sequences: some caveats. Ann Hum Genet 67: 512-524.

S12.**Andrews RM, Kubacka I, Chinnery PF, Lightowlers RN, Turnbull DM, Howell N**. (1999) Reanalysis and revision of the Cambridge reference sequence for human mitochondrial DNA. Nat Genet 23:147.

S13.**Forster P, Harding R, Torroni A, Bandelt HJ.** (1996) Origin and evolution of Native American mtDNA variation: a reappraisal. Am J Hum Genet 59: 935-945.

S14.**Torroni A, Schurr TG, Cabell MF, Brown MD, Nell JV, Larsen M, Smith DG, Vullo CM, Wallace DC.** (1993) Asian affinities and contiental radiation of the four founding Native American mtDNAs. Am J Hum Genet 53: 563-590.

S15.**Kong Q-P, Bandelt H-J, Sun C, Yao Y-G, Salas A, et al.** (2006) Updating the East Asian mtDNA phylogeny a prerequisite for the identification of pathogenic mutations. Hum Mol Genet 15:2076-2086.

S16.**Helgason A, Palsson G, Pedersen HS, Angulalik E, Gunnarsdottir ED, et al.** (2006) MtDNA variation in Inuit populations of Greenland and Canada: migration history and population structure. Am J Phys Anthropol 130:123-134.

S17.**Brown MD, Hosseini SH, Torroni A, Bandelt HJ, Allen JC, et al.** (1998) mtDNA haplgroup X: An ancient link between Europe/Western Asia and North America? Am J Hum Genet 63: 1852-1861.
